# Microstructural, Mechanical, and Adhesive Characterization of Nanofluorapatite-Modified Orthodontic Resin-Modified Glass Ionomer Cement: A Preliminary In Vitro Study

**DOI:** 10.3390/ma19194076

**Published:** 2026-09-23

**Authors:** Wojciech Dobrzyński, Anna Nikodem, Joanna Weżgowiec, Rafał J. Wiglusz, Marcin Mikulewicz

**Affiliations:** 1 Department of Dentofacial Orthopedics and Orthodontics, Division of Facial Abnormalities, Faculty of Dentistry, Wroclaw Medical University, Krakowska 26, 50-425 Wroclaw, Poland; 2 Department of Mechanics, Materials and Biomedical Engineering, Faculty of Mechanical Engineering, Wrocław University of Science and Technology, Łukasiewicza 7/9, 50-371 Wroclaw, Poland; anna.nikodem@pwr.edu.pl; 3 Department of Experimental Dentistry, Faculty of Dentistry, Wroclaw Medical University, Krakowska 26, 50-425 Wroclaw, Poland; joanna.wezgowiec@umw.edu.pl; 4 Institute of Low Temperature and Structure Research, Polish Academy of Sciences, Okólna 2, 50-422 Wroclaw, Poland; r.wiglusz@intibs.pl

**Keywords:** resin-modified glass ionomer cement, nanofluorapatite, micro-computed tomography, BSE-SEM/EDS, instrumented indentation, shear bond strength, orthodontic bonding

## Abstract

Nanofluorapatite (nFAp) was incorporated into GC Fuji Ortho LC resin-modified glass ionomer cement at 2 wt.% and 5 wt.% to assess its influence on microstructure, local mechanical properties, and orthodontic bond strength. The unmodified and nFAp-modified formulations were characterized by micro-computed tomography, backscattered-electron scanning electron microscopy with energy-dispersive X-ray spectroscopy (BSE-SEM/EDS), instrumented Vickers indentation, shear bond strength (SBS) testing, Weibull analysis, and Adhesive Remnant Index (ARI) scoring after debonding of orthodontic molar tubes bonded to human molars. Micro-CT showed higher porosity and resolved pore number density after nFAp incorporation, while pore dimensions changed only modestly. BSE-SEM/EDS showed microscale compositional heterogeneity and a markedly higher Ca signal in the modified materials. Indentation hardness and modulus did not differ significantly among groups. Mean SBS increased from 12.47 ± 2.41 MPa in the reference group to 17.31 ± 2.34 MPa and 16.66 ± 2.60 MPa for 2 wt.% and 5 wt.% nFAp, respectively; both modified groups differed significantly from the reference group but not from each other. ARI distributions were similar among groups. Overall, nFAp incorporation improved resistance to debonding without a corresponding change in local indentation properties, with 2 wt.% nFAp showing the most favorable descriptive overall mechanical profile among the tested formulations.

## 1. Introduction

Fixed orthodontic appliances create additional plaque-retentive areas around brackets, molar tubes, archwires, and adjacent enamel surfaces, making oral hygiene more difficult and promoting biofilm accumulation [1,2]. They may also induce quantitative and qualitative changes in the oral microbiota [1,2]. The resulting increase in cariogenic and acidogenic activity favors enamel demineralization and the development of white spot lesions (WSLs), which represent an early stage of caries characterized by subsurface mineral loss beneath a relatively intact enamel surface [3,4]. WSLs remain a frequent adverse effect of fixed orthodontic treatment. A recent systematic review and meta-analysis reported a pooled prevalence of 55.06% among orthodontic patients and an incidence of 34.2% during treatment [5].

Besides protection of the enamel surface, successful fixed-appliance treatment requires reliable retention of orthodontic attachments. Brackets and molar tubes must remain bonded while subjected to forces generated during orthodontic treatment and normal oral function, yet they must also be removable at the end of treatment without excessive damage to enamel [6]. This balance is particularly relevant for molar tubes because posterior attachments are exposed to substantial occlusal loading. Clinical evidence confirms that molar-tube debonding remains a relevant problem and may delay orthodontic treatment; second molars in particular have been reported to be more susceptible to bond failure [7,8]. Bond failures of orthodontic attachments require additional clinical intervention and may compromise treatment efficiency [7,9,10,11].

Resin-modified glass ionomer cements (RMGICs), including GC Fuji Ortho LC, are attractive orthodontic bonding materials because they combine adhesive properties with fluoride release. RMGICs can act as fluoride reservoirs and release fluoride particularly under low-pH conditions, when the risk of enamel demineralization is increased. Their bonding to enamel also includes a physicochemical component rather than relying exclusively on micromechanical retention [12]. These properties are particularly relevant in patients at increased risk of WSL formation. However, the therapeutic advantages of an orthodontic cement are clinically useful only when adequate mechanical performance and bond strength are maintained. Modification of glass ionomer cements may improve selected biological or physicochemical properties but can also alter their mechanical behavior [13], and studies of fluoride-releasing orthodontic bonding materials therefore emphasize the need to maintain sufficient bond strength [14].

One approach to modifying glass ionomer cements is the incorporation of bioactive nanoscale fillers. Nanofluorapatite (nFAp), a fluoride-containing calcium phosphate with an apatite structure, is of particular interest because it combines calcium and phosphate chemistry with a source of fluoride ions. Previous studies have shown, however, that its effect depends strongly on the base material and modification protocol [15,16]. Moshaverinia et al. incorporated nano-fluorapatite into a conventional glass ionomer cement and reported significantly increased Vickers microhardness and good biocompatibility, while cumulative fluoride release was not significantly different from that of the unmodified cement [15]. In contrast, Lin et al. incorporated nano-fluorapatite or nano-fluorohydroxyapatite into Fuji Ortho LC and obtained substantially greater fluoride release. The unmodified cement showed significantly higher SBS than the nanoparticle-modified formulations, although the nano-fluorapatite-modified cement retained clinically sufficient bond strength [16]. These observations illustrate that an improvement in one property, such as ion release, does not necessarily translate into a corresponding improvement in mechanical performance.

Our previous work on nFAp-modified GC Fuji Ortho LC further demonstrated that the fluoride-release behavior of this material is strongly influenced by the composition and pH of the surrounding medium and that the amount of released fluoride is related to nFAp content [17]. Other nanostructured orthodontic cements have also shown that bioactive functionality can be combined with acceptable bond strength. Yi et al. developed an RMGIC containing nano-CaF_2_ together with the antibacterial monomer DMAHDM (Dimethylaminohexadecyl methacrylate), which enhanced antibacterial and remineralization properties without compromising bracket–enamel SBS [18]. In a related study, an nCaF_2_-containing orthodontic cement showed sustained fluoride recharge and re-release while maintaining SBS comparable to a commercial RMGIC [19]. Together, these studies demonstrate the potential of calcium- and fluoride-containing nanophases in orthodontic cements, while also showing that their effects are highly dependent on formulation and concentration.

Despite these findings, the structural consequences of incorporating nFAp into orthodontic RMGICs and their relationship with mechanical and adhesive performance remain insufficiently characterized. In particular, information is limited on whether changes in nFAp content are accompanied by changes in internal porosity and pore morphology, local elemental composition, indentation response, and resistance of the bonded orthodontic system to debonding. This is important because increasing the amount of a nanoparticle additive does not necessarily produce a proportional improvement in mechanical performance [13,15,16]. A combined assessment of material microstructure and bonding behavior is therefore required to determine how nFAp modification influences GC Fuji Ortho LC and whether the observed effects occur within the cement itself or become apparent mainly at the level of the bonded tooth–cement–orthodontic attachment system.

Accordingly, the aim of this in vitro study was to evaluate the effect of 2 wt.% and 5 wt.% nFAp incorporation on the structural, mechanical, and adhesive properties of GC Fuji Ortho LC used for bonding orthodontic molar tubes VIPER™ (G&H Orthodontics, Franklin, IN, USA). Internal porosity and pore morphology were assessed by micro-computed tomography, while BSE-SEM/EDS (backscattered electron scanning electron microscopy and energy-dispersive X-ray spectroscopy) was used to characterize the internal microstructure and local elemental composition. The local mechanical response of the cement was evaluated by instrumented Vickers indentation, and orthodontic bonding performance was assessed by shear bond strength testing, Weibull analysis, and Adhesive Remnant Index scoring after debonding (Figure 1).

Based on this rationale, the following research hypotheses were formulated: H1, incorporation of 2 wt.% and 5 wt.% nFAp would alter the internal microstructure and local elemental composition of GC Fuji Ortho LC compared with the unmodified reference material; H2, nFAp incorporation would significantly alter the indentation-derived mechanical properties of the cement; H3, nFAp incorporation would significantly increase the shear bond strength of orthodontic molar tubes compared with the unmodified reference material; and H4, nFAp incorporation would significantly alter the ARI score distribution after debonding.

## 2. Materials and Methods

The experimental study was conducted in two consecutive stages. The first stage focused on the physicochemical, microstructural, and mechanical characterization of the material itself. The nFAp-modified GC Fuji Ortho LC formulations had been developed previously; therefore, the aim of this stage was to determine how incorporation of 2 wt.% and 5 wt.% nFAp affected the internal structure and properties of the glass-ionomer cement. This stage comprised micro-computed tomography analysis of porosity and pore morphology, BSE-SEM/EDS characterization of the internal microstructure and elemental composition, and instrumented indentation measurements of hardness and elastic modulus.

The second stage evaluated the modified cement in its intended orthodontic application. The investigated formulations were used for bonding orthodontic molar tubes to human enamel, followed by mechanical assessment of shear bond strength and evaluation of the adhesive failure pattern after debonding. Thus, the experimental workflow progressed from characterization of the modified material itself to assessment of its functional performance as an orthodontic bonding cement.

Figure 1 was conceptually designed and assembled by the authors using BioRender. ChatGPT (OpenAI, image generation/editing functionality, accessed August 2026) was subsequently used solely to assist with refinement of the visual presentation and image quality of the figure; the scientific content, experimental workflow, labels, and interpretation were defined and verified by the authors.

### 2.1. Materials

The base material used in this study was the commercial light-curing two-component orthodontic cement GC Fuji Ortho^®^ LC (GC Corporation, Tokyo, Japan). Three formulations were investigated: unmodified GC Fuji Ortho LC (REF), GC Fuji Ortho LC modified with 2 wt.% nanofluorapatite (nFAp), and GC Fuji Ortho LC modified with 5 wt.% nFAp. The nFAp concentration was calculated relative to the mass of the cement powder, in accordance with the previously established preparation protocol.

### 2.2. Nanofluorapatite Synthesis and Cement Modification

Nanocrystalline fluorapatite powder was synthesized at the Institute of Low Temperature and Structure Research, Polish Academy of Sciences, Wrocław, Poland, using a co-precipitation method previously described by Dobrzyński et al. [17]. Calcium nitrate tetrahydrate (Ca(NO_3_)_2_·4H_2_O), diammonium hydrogen phosphate ((NH_4_)_2_HPO_4_), and ammonium fluoride (NH_4_F) were used as precursor materials, while aqueous ammonia (NH_4_OH) was used for pH adjustment. Stoichiometric amounts of the precursor compounds were dissolved in deionized water and mixed on a magnetic stirring plate at 100 °C for 1.5 h while maintaining the reaction pH at approximately 10. The resulting precipitate was washed and centrifuged at least three times until neutral pH was obtained, dried at 70 °C for 24 h, and subsequently heat-treated at 450 °C for 6 h.

For the modified formulations, nFAp was incorporated into the GC Fuji Ortho LC cement powder at concentrations of 2 wt.% or 5 wt.% relative to the mass of the cement powder. The required amount of nFAp powder was added directly to the cement powder and mixed manually for approximately 5 min using a circular mixing motion. This procedure was selected on the basis of preliminary methodological studies, which showed that circular mixing resulted in greater material homogeneity and lower porosity than bidirectional right–left mixing. The circular mixing procedure was therefore used consistently throughout the final experimental series.

### 2.3. Cylindrical Specimen Preparation

Cylindrical specimens measuring 6 mm in diameter and 2.5 mm in height were prepared for subsequent microstructural and mechanical characterization. The prepared cement was placed in a dedicated mould and polymerized using an Elipar II LED curing unit (3M ESPE, St. Paul, MN, USA), emitting light in the wavelength range of 400–515 nm at an irradiance of up to 800 mW/cm^2^. Each specimen was light-cured for 20 s. After preparation, the cylindrical specimens were stored under dry conditions at 23 ± 2 °C for 24 h before microstructural and mechanical testing. No liquid storage medium, thermocycling, or other artificial ageing procedure was used for these specimens.

### 2.4. Micro-Computed Tomography Analysis

The internal microstructure and porosity of the investigated cement formulations were evaluated using X-ray micro-computed tomography (micro-CT) with a SkyScan 1172 system (Bruker, Kontich, Belgium). One physical specimen from each formulation was examined (*n* = 1 per formulation). All specimens were scanned using the same general acquisition protocol, although the X-ray source settings and rotation step differed slightly between specimens. The REF specimen was scanned at 100 kV and 100 µA with a rotation step of 0.400°, the 2 wt.% nFAp specimen at 89 kV and 112 µA with a rotation step of 0.430°, and the 5 wt.% nFAp specimen at 100 kV and 100 µA with a rotation step of 0.430°. Scanning was performed using 2 × 2 camera binning, an Al + Cu filter, an exposure time of 1150 ms, two-frame averaging, random movement level 3, and step-and-shoot acquisition. Projection images were acquired as 16-bit TIFF files. The reconstructed voxel size was 5.93 µm.

Image reconstruction was performed using NRecon software (version 1.7.4.6, Bruker) in standard reconstruction mode. Smoothing was set to 0, ring-artifact correction to 18, and beam-hardening correction to 0%. A Hamming filter (α = 0.54) was applied.

Quantitative analysis was performed using Dragonfly 3D World software (version 2025.1, Build 2081; Object Research Systems, Montreal, QC, Canada). For each formulation, three spatially separated regions of interest (ROIs), each approximately 2 mm in diameter, were analyzed to assess local spatial variability within the specimen. In addition, the reconstructed specimen was analyzed as a whole to provide complementary whole-specimen measurements.

The cement phase was segmented using the Upper Otsu method, followed by identification of internal voids using the Porosity Segmentation with AO Fields algorithm with an openness setting of 95%. Volumetric porosity was calculated as the ratio of the total segmented pore volume to the total analyzed specimen or ROI volume.

Individual pores were separated by three-dimensional connected-component analysis using 26-connectivity. Components with a minimum Feret diameter of at least three voxels (17.80 µm) were classified as resolved pores. Resolved pore number density was calculated as the number of resolved pores per unit analyzed volume and expressed in mm^−3^. The resolved pore-volume fraction was calculated as the cumulative volume of resolved pores divided by the total segmented pore volume and expressed as a percentage. Equivalent spherical diameter (ESD) and minimum, mean, and maximum Feret diameters were determined for the resolved pore population. The 17.80 µm size criterion was applied only to connected-component-based pore counting and morphometric analysis and did not affect the voxel-based calculation of total volumetric porosity.

### 2.5. BSE-SEM and EDS Analysis

The microstructure of the investigated cement formulations was further examined using a Phenom Pharos G2 Desktop FEG-SEM scanning electron microscope equipped with an EDS detector (Thermo Fisher Scientific, Eindhoven, The Netherlands). SEM/EDS analysis was performed as a complementary method to micro-CT because the applied micro-CT voxel size did not allow direct identification or discrimination of the nanofluorapatite phase.

Cylindrical specimens from REF, 2 wt.% nFAp, and 5 wt.% nFAp groups were mechanically fractured to expose fresh internal surfaces. The fractured surfaces were examined directly, without additional grinding or polishing, to assess the internal morphology of the cement matrix and compositionally distinct regions potentially associated with the incorporated nFAp.

The specimens were examined without conductive coating under low-vacuum conditions at a chamber pressure of approximately 60 Pa and an accelerating voltage of 15 kV. Backscattered-electron images were acquired using the full backscattered-electron detector configuration (BSD Full), providing compositional contrast within the cement matrix.

EDS analysis was performed to characterize the local elemental composition of the fractured surfaces, with particular attention to Ca, P, and F. Quantitative spot analysis included four REF specimens, three specimens containing 2 wt.% nFAp, and three specimens containing 5 wt.% nFAp. Three EDS spot measurements were performed for each specimen, and the measurements were averaged to obtain a specimen-level value for subsequent group-level descriptive comparison. One additional specimen from the 5 wt.% nFAp group (specimen 87) was excluded from quantitative EDS comparison because it had been coated with Au and was therefore not directly comparable with the uncoated specimens.

BSE morphology and EDS composition were evaluated jointly. Because the unmodified glass-ionomer cement itself contains fluoride- and silicate-containing inorganic components, the presence of individual elements was not considered sufficient evidence for direct identification of the nFAp phase. Accordingly, EDS was used to assess local compositional differences and elemental distribution rather than to identify individual nFAp particles unequivocally.

### 2.6. Instrumented Vickers Indentation Testing

The mechanical properties of the investigated cement formulations were evaluated by instrumented indentation using a Micro Combi Tester MCT (CSM Instruments, Peseux, Switzerland) equipped with a Vickers diamond indenter. Three cylindrical specimens from each formulation (REF, 2 wt.% nFAp, and 5 wt.% nFAp) were used for the measurements.

Before testing, the cross-sectional surfaces of the specimens were prepared using a FORCIPOL 102 metallographic grinding machine (Metkon Instruments Inc., Bursa, Turkey). The surfaces were wet-ground under continuous water irrigation using 2500-grit abrasive paper to obtain flat and smooth measurement surfaces. No additional polishing procedure was performed. After grinding, the surfaces were gently cleaned with compressed air to remove residual water and loose surface debris. Prior to indentation, the surfaces were visually inspected, and measurement sites were selected away from visible pores, cracks, scratches, specimen edges, and other surface defects.

Indentation tests were performed under load-controlled conditions with a maximum load of 400 mN. The loading and unloading rates were both 800 mN/min, corresponding to approximately 30 s for each loading and unloading segment. The maximum load was maintained for 20 s before unloading. Contact with the specimen surface was detected at a load of 25 mN, and force–displacement data were recorded at an acquisition frequency of 10 Hz. A Poisson’s ratio of 0.30 was assumed for calculation of the indentation modulus.

Three indentations were performed on each specimen at spatially separated locations. Adequate spacing was maintained between adjacent indentations to minimize interaction between the corresponding deformation fields. Measurement quality was verified by inspection of the indentation sites and the recorded load–displacement curves.

Martens hardness (HM), indentation hardness (HIT), Vickers indentation hardness (HVIT), indentation modulus (EIT), and the elastic-to-total indentation work ratio (ηIT) were determined from the indentation measurements. HM was determined from the applied load and indentation depth according to the geometry of the Vickers indenter. EIT was calculated from the unloading segment according to the Oliver–Pharr method, using the 40–98% unloading range for curve fitting. The elastic-to-total indentation work ratio (ηIT) was calculated as the ratio of elastic indentation work to total indentation work and expressed as a percentage.

For each specimen, the three indentation measurements were averaged to obtain a single specimen-level value. The specimen mean was subsequently used as the statistical unit for comparison among the three cement formulations.

### 2.7. Specimen Preparation and Bonding

Thirty healthy human molars, free of caries, restorations, cracks, and other visible structural defects, and extracted for orthodontic indications, were used for the shear bond strength assessment. Following extraction, the teeth were cleaned of residual soft tissues and stored in a 0.05% aqueous thymol solution at 4 ± 1 °C in darkness for no longer than 3 months. Twenty-four hours before bonding, the teeth were thoroughly rinsed and transferred to distilled water. The extracted teeth constituted fully anonymized residual biological material obtained following routine clinical treatment at the Department of Pediatric Dentistry and Preclinical Dentistry, Wroclaw Medical University, Poland. The extractions were performed solely for clinical indications unrelated to the present study. No personal identifiers or clinical data were collected or linked to the specimens. According to the institutional and regulatory framework applicable to research using fully anonymized residual biological material, formal ethics committee approval and additional donor consent were not required. The teeth were allocated to three experimental groups (*n* = 10 per group) according to the cement formulation used for bonding: unmodified GC Fuji Ortho^®^ LC (REF), GC Fuji Ortho^®^ LC containing 2 wt.% nFAp, and GC Fuji Ortho^®^ LC containing 5 wt.% nFAp. The group size of 10 specimens was selected with reference to previous in vitro orthodontic debonding studies using comparable SBS protocols [6,20,21] and the availability of eligible extracted human molars. No formal a priori power calculation was performed.

Before bonding, the buccal enamel surface was cleaned and etched with 37% orthophosphoric acid for 20 s, thoroughly rinsed with distilled water, and dried until a characteristic chalky-white appearance was obtained. GC Fuji Ortho^®^ LC was prepared by mixing the powder and liquid at the manufacturer-recommended ratio of 3.0 g powder to 1.0 g liquid. Depending on the experimental group, unmodified cement powder or cement powder containing 2 wt.% or 5 wt.% nFAp was used.

VIPER™ orthodontic molar tubes (G&H Orthodontics, Franklin, IN, USA) were coated with a thin layer of the prepared cement and positioned centrally on the buccal surface of the tooth crown. Excess cement was carefully removed with a dental probe, after which the material was light-cured using an LED curing unit for 20 s at an approximate distance of 2 cm from the bonded surface. Representative images of an orthodontic molar tube bonded to the tooth surface are shown in Figure 2. Following bonding and photopolymerization, the specimens were stored in distilled water at 37 °C for 24 h before SBS testing.

### 2.8. Shear Bond Strength Testing

The mechanical performance of the tooth–cement–orthodontic tube complex was evaluated using a shear bond strength (SBS) test. The testing procedure was based on protocols previously developed and applied by our group for mechanical debonding of orthodontic attachments [20,21] and was consistent with the general principles of DIN 13990:2017-04 [22], Dentistry—Test Methods for Shear Bond Strength of Adhesives for Orthodontic Attachments.

Each tooth was embedded in Duracryl™ Plus self-polymerizing acrylic resin (SpofaDental, Jičín, Czech Republic) to ensure rigid fixation during testing. The specimens were positioned so that the loading direction was tangential to the buccal enamel surface and aligned with the long axis of the bonded orthodontic tube (Figure 2). Mechanical testing was performed using an MTS 858 MiniBionix^®^ testing system (MTS Systems, Eden Prairie, MN, USA). A flat-ended loading punch was positioned adjacent to the orthodontic tube and advanced at a constant displacement rate of 1 mm/min until complete debonding occurred.

The maximum debonding force was recorded in newtons. For each orthodontic molar tube, the dimensions of the bonding base were measured individually from calibrated stereomicroscopic images, and the bonded surface area was calculated from these measurements. An individual bonded surface area was therefore used for each specimen rather than a single nominal value for all tubes. SBS was calculated by dividing the maximum debonding force by the individually determined bonded surface area and was expressed in MPa.

### 2.9. Adhesive Remnant Index

The Adhesive Remnant Index (ARI) was evaluated for all specimens included in the final shear bond strength analysis (*n* = 28: REF, *n* = 10; 2 wt.% nFAp, *n* = 9; 5 wt.% nFAp, *n* = 9). Following debonding, the enamel surfaces were examined using a SteREO Discovery.V20 stereomicroscope (Carl Zeiss Microscopy GmbH, Jena, Germany) at 0.63× magnification. Digital images were acquired for each specimen and used to assess the amount and distribution of residual cement on the enamel surface.

ARI classification was based exclusively on the amount of cement remaining on the enamel surface. Images of the corresponding orthodontic molar tube bases were used for descriptive and illustrative purposes only and were not considered in ARI scoring.

The residual cement pattern was classified using the Adhesive Remnant Index (ARI) according to previously described criteria [23,24,25]. The following four-point scale was applied:

- ARI 0: no cement remaining on the enamel surface;
- ARI 1: less than 50% of the cement remaining on the enamel surface;
- ARI 2: more than 50% of the cement remaining on the enamel surface;
- ARI 3: all cement remaining on the enamel surface, with a visible imprint of the orthodontic tube mesh.

### 2.10. Statistical Analysis

Statistical analyses were performed using Statistica (TIBCO Software Inc., USA) and GraphPad Prism 9.0 (GraphPad Software Inc., San Diego, CA, USA). Statistical significance was set at *p* < 0.05.

No formal a priori power calculation was performed. To characterize the inferential sensitivity associated with the achieved sample sizes, a sensitivity analysis was additionally considered using α = 0.05 and a target power of 80% (G*Power software (ver. 3.1.9.7, Heinrich-Heine-Universität Düsseldorf)). For the indentation analysis, with three groups ans three independent specimens per formulation (total *n* = 9), only very large standardized omnibus effects could be detected reliably. For conventional one-way ANOVA, the minimum detectable effect was approximately Cohen’s f = 1.36. For the SBS analysis (*n* = 28), the corresponding minimum detectable omnibus effect was approximately f = 0.62. The ARI analysis involved sparse ordinal categorical data and an exact test; therefore, its nonsignificant result was interpreted cautiously rather than as evidence of equivalence among groups.

For micro-CT analysis, one physical specimen was examined per cement formulation; therefore, no inferential comparisons were performed between formulations. Results from the three spatially separated Ø2 mm regions of interest were expressed as mean ± standard deviation (SD), with SD representing within-specimen spatial variability. Pore-size parameters pooled across the analyzed regions were summarized as mean ± SD or median with the first and third quartiles (Q1–Q3), as appropriate.

For EDS analysis, three spot measurements were averaged within each specimen to obtain a single specimen-level value. Group results were summarized as mean ± SD. Because EDS was used as a semiquantitative complementary method and the number of independent specimens was limited, no inferential statistical comparisons were performed between formulations.

For instrumented indentation, valid indentation measurements were averaged within each specimen, and the specimen mean was used as the statistical unit (*n* = 3 specimens per formulation). Results were expressed as mean ± SD, and differences among REF, 2 wt.% nFAp, and 5 wt.% nFAp formulations were evaluated using Welch’s one-way ANOVA.

For shear bond strength (SBS), only tests with a complete load–displacement record and an unambiguously identified debonding event were included. SBS data were summarized as mean ± SD, median, and 95% confidence interval (CI). Normality was assessed using the Shapiro–Wilk test and homogeneity of variance using Levene’s test. When the assumptions for parametric analysis were satisfied, differences among the three formulations were evaluated using one-way ANOVA followed by Tukey’s HSD test for pairwise comparisons. Effect size was expressed as eta-squared (η^2^).

SBS distributions were additionally characterized using a two-parameter Weibull model. The Weibull shape parameter (β) and scale parameter (η) were estimated by maximum likelihood estimation (MLE), with the location parameter fixed at zero. The shape parameter β was used to describe the dispersion of SBS values, with higher β values indicating a narrower distribution, whereas η represented the characteristic shear bond strength.

Adhesive Remnant Index (ARI) scores were treated as ordinal categorical data and reported as frequencies and percentages. Differences in ARI score distribution among the three formulations were evaluated using the Fisher–Freeman–Halton exact test because of the small, expected frequencies in the contingency table.

## 3. Results

### 3.1. Micro-Computed Tomography Analysis

Micro-CT analysis revealed descriptive differences in the internal porosity of the investigated cement formulations. In the three analyzed Ø2 mm regions, volumetric porosity was approximately 61% higher in the 2 wt.% nFAp formulation and 76% higher in the 5 wt.% nFAp formulation than in REF (Table 1).

The same ordering was observed in the whole-specimen measurements, for which porosity was approximately 24% and 32% higher in the 2 wt.% and 5 wt.% nFAp formulations, respectively, compared with the reference group. Thus, both the local Ø2 mm analysis and the whole-specimen assessment showed a progressive increase in the measured porosity values with increasing nFAp content. Representative three-dimensional reconstructions are shown in Figure 3.

The increase in measured volumetric porosity was accompanied by a higher density of resolved pores. In the Ø2 mm regions, resolved pore number density was approximately 23% higher in the 2 wt.% nFAp formulation and 29% higher in the 5 wt.% nFAp formulation than in the reference group. Whole-specimen analysis showed the same ordering, with corresponding increases of approximately 14% and 21%.

The resolved pore-volume fraction showed a similar pattern. Relative to the reference group, values obtained from the Ø2 mm regions were approximately 9.8% higher for the 2 wt.% nFAp formulation and 10.8% higher for the 5 wt.% nFAp formulation. The corresponding differences in the whole-specimen analysis were smaller, amounting to approximately 2.9% and 3.5%, respectively.

In contrast, the characteristic dimensions of the resolved pores showed only modest differences among the formulations (Table 2). In the pooled Ø2 mm regions, median equivalent spherical diameter (ESD) was approximately 5.0% higher in the 2 wt.% nFAp formulation and 4.3% higher in the 5 wt.% nFAp formulation than in the reference group. Whole-specimen median ESD differed by only approximately +2.1% and +2.7%, respectively.

Mean ESD also differed only modestly among the formulations. Compared with the reference group, mean ESD in the pooled Ø2 mm regions was approximately 8.1% higher in the 2 wt.% nFAp formulation and 8.3% higher in the 5 wt.% nFAp formulation. Changes in Feret dimensions were similarly limited. Median Feret Mean was approximately 5.4% and 5.0% higher, whereas median Feret Max was approximately 5.9% and 3.0% higher in the 2 wt.% and 5 wt.% nFAp formulations, respectively, than in the reference group. These differences were considerably smaller than the corresponding differences in volumetric porosity and resolved pore number density.

Overall, micro-CT analysis showed a progressive increase in the measured volumetric porosity and resolved pore number density with increasing nFAp content. In the Ø2 mm regions, porosity was approximately 61% and 76% higher, while resolved pore number density was approximately 23% and 29% higher in the 2 wt.% and 5 wt.% nFAp formulations, respectively, than in the reference group. In contrast, differences in characteristic pore dimensions were generally below approximately 10%. The observed structural differences were therefore associated predominantly with greater pore abundance and pore-volume contribution rather than with marked enlargement of individual resolved pores.

Analysis of the reconstructed grayscale distributions did not reveal a distinct high-attenuation population that could be reliably attributed to nFAp. Consequently, individual nFAp particles or nFAp-rich regions could not be unequivocally identified or quantitatively segmented by micro-CT at the applied voxel size. Further characterization of the internal material microstructure and local elemental composition was therefore performed using BSE-SEM/EDS.

### 3.2. SEM/EDS Analysis

BSE-SEM examination revealed a heterogeneous microstructure in all investigated cement formulations, characterized by irregular inorganic particles dispersed within the cement matrix and locally occurring voids. No morphologically distinct particle population that could be unequivocally assigned to nFAp was identified. The nFAp-modified specimens showed local variations in particle distribution and compositional contrast, indicating microscale heterogeneity within the cement structure. Representative BSE images and elemental maps are shown in Figure 4.

EDS analysis identified the principal inorganic constituents of the cement, including F, Al, Si, P, Ca, and Sr. The semiquantitative comparison included four REF specimens, three 2 wt.% nFAp specimens, and three 5 wt.% nFAp specimens; specimen-level mean values were used for descriptive group comparison.

The most pronounced compositional difference was observed for Ca (Table 3). Compared with REF, the mean Ca signal was approximately 178% higher in the 2 wt.% nFAp formulation and 168% higher in the 5 wt.% nFAp formulation. Thus, both nFAp-modified formulations exhibited an approximately 2.7–2.8-fold higher Ca signal than the unmodified cement.

In contrast, P showed only minor differences relative to REF, amounting to approximately +3.7% for the 2 wt.% nFAp formulation and −0.2% for the 5 wt.% formulation. Fluorine showed no consistent trend with increasing nFAp content, being approximately 10.1% lower in the 2 wt.% formulation and 1.7% higher in the 5 wt.% formulation than in REF.

Local EDS measurements also revealed considerable compositional heterogeneity within the nFAp-modified specimens. Particularly pronounced point-to-point variation was observed in specimens 97 and 86, whereas specimen 104 showed a more uniform Ca distribution across the analyzed regions. These observations were consistent with the heterogeneous elemental distributions visible in the EDS maps.

Overall, semiquantitative EDS showed a substantially higher Ca signal in both nFAp-modified formulations than in REF, whereas P and F did not show a corresponding monotonic change with increasing nFAp content. The local variability in Ca and P further indicated a heterogeneous microscale distribution of the inorganic components. Although the higher Ca signal was consistent with incorporation of the Ca-containing nFAp additive, no distinct Ca–P–F compositional signature was identified that would allow individual nFAp particles or agglomerates to be unequivocally distinguished. Accordingly, H1 was accepted within the descriptive scope of the microstructural analyses.

### 3.3. Instrumented Indentation Analysis

Instrumented indentation measurements showed modest changes in hardness-related parameters following nFAp incorporation, whereas indentation modulus remained essentially unchanged (Table 4). After quality control, individual indentation measurements were averaged within each specimen, and statistical comparisons were performed at the specimen level (*n* = 3 per formulation).

Compared with the unmodified reference group, Martens hardness (HM) increased by approximately 7.4% in the 2 wt.% nFAp formulation and 8.5% in the 5 wt.% nFAp formulation. A slightly greater increase was observed for indentation hardness (H_IT_), which was approximately 10.8% higher for 2 wt.% nFAp and 11.1% higher for 5 wt.% nFAp. Vickers indentation hardness (HV_IT_) showed the same relative pattern, increasing by approximately 10.8% and 11.1%, respectively. Thus, both nFAp-modified formulations exhibited consistently higher mean hardness values than the reference group, although the magnitude of the increase was similar for the two nFAp concentrations.

In contrast, indentation modulus (E_IT_) was largely unaffected by nFAp incorporation. Relative to REF, E_IT_ differed by only −1.7% in the 2 wt.% nFAp formulation and −0.3% in the 5 wt.% formulation. The elastic-to-total indentation work ratio (η_IT_) also remained stable, showing changes of only approximately +0.5% and +2.4%, respectively. These results indicate that the numerical effect of nFAp incorporation was more pronounced for hardness-related parameters than for elastic stiffness or elastic recovery.

Despite the numerical increase in hardness, Welch’s one-way ANOVA showed no statistically significant differences among the formulations for HM, H_IT_, E_IT_, HV_IT_, or η_IT_ (all *p* > 0.05). Accordingly, no post-hoc pairwise comparisons were performed.

Overall, nFAp incorporation was associated with an approximately 7–11% increase in hardness-related parameters, while changes in EIT remained below 2% and changes in η_IT_ below 3%. The mechanical response therefore suggests a modest increase in indentation hardness without a corresponding change in elastic stiffness; however, these differences were not statistically significant. Accordingly, H2 was not supported by the present data.

### 3.4. Shear Bond Strength

Valid shear bond strength (SBS) measurements were obtained for 28 specimens: 10 in REF group and 9 in each of the nFAp-modified groups. The remaining specimens were excluded because of damage before testing or instability and slippage associated with individual tooth geometry during mechanical loading.

SBS differed significantly among the investigated cement formulations (Table 5). Compared with the unmodified reference cement, mean SBS increased by approximately 38.8% following incorporation of 2 wt.% nFAp and by 33.5% following incorporation of 5 wt.% nFAp. The highest mean SBS was observed for the 2 wt.% nFAp formulation; however, the difference between the two nFAp-modified groups was only approximately 3.9%.

The SBS data satisfied the assumptions for parametric analysis. Shapiro–Wilk tests showed no significant departure from normality in any group (all *p* > 0.11), and Levene’s test indicated homogeneity of variances (*p* = 0.929). One-way ANOVA demonstrated a significant effect of cement formulation on SBS (F (2,25) = 11.044, *p* = 0.00037), with a large effect size (η^2^ = 0.469). Tukey’s post-hoc analysis showed that SBS was significantly higher for both the 2 wt.% nFAp (*p* = 0.0007) and 5 wt.% nFAp (*p* = 0.0029) formulations compared with REF. No statistically significant difference was detected between the two nFAp-modified formulations (*p* = 0.8387).

Weibull analysis showed a corresponding shift toward higher characteristic bond strength in both nFAp-modified groups. Relative to REF, the Weibull scale parameter (η) increased by approximately 36.1% for the 2 wt.% nFAp formulation and by 32.0% for the 5 wt.% nFAp formulation. The Weibull shape parameter (β) increased by approximately 36.8% and 18.7%, respectively. The 2 wt.% nFAp formulation exhibited the highest values of both β and η; its β value was approximately 15.2% higher than that of the 5 wt.% formulation, corresponding to a narrower distribution of SBS values. Overall, both nFAp-modified formulations showed significantly higher SBS than the unmodified cement, whereas increasing the nFAp concentration from 2 wt.% to 5 wt.% did not result in a further statistically significant increase. Accordingly, H3 was accepted.

### 3.5. Adhesive Remnant Index

The distribution of Adhesive Remnant Index (ARI) scores did not differ significantly among the investigated cement formulations (Fisher–Freeman–Halton exact test, *p* = 0.761) (Table 6).

ARI score 1 was the most frequently observed failure pattern in all three groups, accounting for 50.0% of specimens in REF group and 55.6% in both nFAp-modified groups. In REF group, ARI scores 0 and 2 were observed in 30.0% and 20.0% of specimens, respectively. In the 2 wt.% nFAp group, ARI score 0 occurred in 33.3% of specimens, whereas ARI score 2 was observed in 11.1%. In the 5 wt.% nFAp group, the corresponding proportions were 11.1% and 33.3%, respectively.

ARI scores ≤ 1 were observed in 80.0% of REF specimens, 88.9% of specimens in the 2 wt.% nFAp group, and 66.7% of specimens in the 5 wt.% nFAp group. ARI score 3 was not observed in any of the analyzed specimens. Overall, despite some differences in the relative frequencies of individual ARI scores, no statistically significant change in the ARI score distribution was detected following nFAp incorporation. Representative stereomicroscopic images of the enamel surfaces and corresponding orthodontic molar tube bases after debonding are shown in Figure 5. Accordingly, H4 was not supported by the present data.

## 4. Discussion

The present study showed that the effect of nFAp incorporation into GC Fuji Ortho LC was more pronounced at the level of the bonded tooth–cement–orthodontic tube system than in the local mechanical response of the cement matrix itself. The modified formulations showed higher porosity and resolved pore number density, together with microscale compositional heterogeneity and a markedly higher local Ca signal. At the same time, hardness-related parameters increased only modestly, and the indentation modulus remained essentially unchanged, with no statistically significant differences among the formulations. In contrast, SBS increased after incorporation of both nFAp concentrations. This improvement in bond strength was not accompanied by a significant change in ARI distribution. These findings indicate that the mechanical consequences of nFAp modification cannot be described simply as reinforcement of the bulk cement matrix. Accordingly, H1 and H3 were accepted, although H1 was supported within the descriptive scope of the microstructural analyses, whereas H2 and H4 were not supported by the present data.

Micro-CT revealed a clear descriptive trend toward higher porosity with increasing nFAp content, whereas characteristic pore dimensions changed only modestly. The structural effect of nFAp incorporation therefore appeared to involve mainly an increase in the abundance and overall volume contribution of pores rather than the formation of substantially larger pores. Because only one physical specimen per formulation was examined by micro-CT, these differences should be regarded as descriptive and cannot establish a statistically supported concentration-dependent relationship.

The micro-CT data therefore do not support a simple model in which nanoparticle incorporation improves mechanical behavior by increasing particle packing and reducing structural defects. The response of glass-ionomer materials to nanofiller incorporation depends on filler type and concentration, dispersion within the matrix, interaction between the added phase and the original cement components, and the preparation procedure itself [26,27,28]. Previous studies have also shown that increasing the amount of a particulate additive does not necessarily produce a proportional mechanical benefit [28,29,30,31,32,33,34,35,36]. At higher filler contents, increased viscosity, impaired homogenization, local agglomeration, or air entrapment may offset potential reinforcement [28,29,30,31,32,33,34,35,37]. Nanoparticle incorporation may also affect the handling and setting characteristics of glass-ionomer cements. In an RMGIC modified with amorphous calcium phosphate nanoparticles, Karimi et al. reported a concentration-dependent reduction in working time and changes in setting behavior, with the reduced workability attributed in part to the high specific surface area of the nanoparticles and the resulting increase in cement viscosity [37]. However, viscosity, handling properties, and setting kinetics were not directly evaluated in the present study; therefore, their possible contribution to the similar SBS values observed for the 2 wt.% and 5 wt.% nFAp formulations remains hypothetical. In the present material, the progressive increase in measured porosity with increasing nFAp content is consistent with such a possibility, although the available micro-CT dataset does not allow the origin of the pores to be established directly.

BSE-SEM/EDS provided complementary information on the internal structure of the modified cements. No morphologically distinct particle population could be unequivocally identified as nFAp in the BSE images. Nevertheless, semiquantitative EDS showed an approximately 2.7–2.8-fold higher Ca signal in both modified formulations than in REF. In comparison, P remained broadly similar among the groups and F did not show a monotonic increase with nFAp concentration. Considerable point-to-point variability in Ca and P was also observed within individual modified specimens. Thus, nFAp incorporation was associated with a clear change in local elemental composition, but not with a uniform Ca–P–F signature that would permit direct identification of individual nFAp particles or agglomerates. The elevated Ca signal should therefore be regarded as consistent with incorporation of the Ca-containing nFAp phase, but not as direct evidence of discrete nFAp particles.

Despite these structural and compositional changes, the local mechanical response measured by instrumented indentation remained relatively stable. Martens hardness, indentation hardness, and Vickers indentation hardness were numerically approximately 7–11% higher in the nFAp-modified formulations, but none of these differences reached statistical significance. The indentation modulus differed by less than 2% from REF. Moshaverinia et al. reported increased microhardness after incorporation of nano-fluorapatite into a conventional glass-ionomer cement [15], whereas Lin et al. investigated nano-fluorapatite and nano-fluorohydroxyapatite modification of Fuji Ortho LC and showed that the effect of the added phase depended strongly on the specific formulation and property examined [16]. Differences between studies are therefore not unexpected as the indentation response of light-cured dental materials may depend on material composition, filler characteristics, filler–matrix interactions, and curing conditions [15,16,38]. In the present system, 2–5 wt.% nFAp did not produce a statistically demonstrable increase in the local stiffness of the cement matrix.

The coexistence of increased porosity and largely unchanged indentation properties is not necessarily contradictory. Instrumented indentation probes a very small material volume, and in the present study the indentation sites were intentionally selected away from visible pores, cracks, scratches, and other surface defects. The resulting values therefore characterize relatively intact local regions of the cement matrix. Debonding, in contrast, is a failure process involving the entire cement layer, both interfaces, the retentive tube base, and structural discontinuities distributed throughout the bonded area. Pores and other defects may act as local stress concentrators and sites of crack initiation and may therefore have a greater influence on fracture and debonding than on a localized indentation measurement. The two methods consequently probe different aspects and spatial scales of the material response.

The strongest functional effect of nFAp incorporation was observed in SBS. Both nFAp-modified formulations showed significantly higher SBS than REF, whereas increasing the nFAp concentration from 2 wt.% to 5 wt.% did not produce a further significant improvement. This confirms that the effect of nFAp on orthodontic bonding is not simply proportional to the amount of additive incorporated into the cement. Previous studies of calcium-phosphate-containing orthodontic materials have likewise shown that the influence of bioactive fillers on bond strength is strongly dependent on the material system and modification protocol [6,15,16,39,40,41,42]. In particular, Lin et al. reported that incorporation of nano-fluorapatite or nano-fluorohydroxyapatite into Fuji Ortho LC altered functional properties while maintaining clinically useful bond strength but did not produce a straightforward increase in SBS relative to the unmodified cement [16]. The present results therefore extend previous observations by showing that, under the preparation and testing conditions used here, nFAp incorporation can significantly increase resistance to debonding.

The increase in SBS should not be attributed automatically to reinforcement of the cement matrix. If the main mechanism were a substantial increase in bulk hardness or stiffness, a corresponding change in HIT, HM, or EIT would be expected, whereas no significant differences were observed. The improvement is therefore more likely to involve the behavior of the complete bonded assembly. Potential contributions may include changes in local matrix composition, stress transfer within the cement layer, mechanical interlocking with the tube base, or interactions at the cement–enamel interface [43]. RMGICs are capable of interacting chemically with dental hard tissues and are also characterized by fluoride release [12,13,14]. Previous work on the same nFAp-modified Fuji Ortho LC system showed that nFAp incorporation modified fluoride-release behavior under different pH conditions [17], demonstrating that the additive affects the physicochemical behavior of the material. However, the cement–enamel interface was not examined directly in the present study, and a specific interfacial mechanism responsible for the increase in SBS cannot be established from the current data.

The lack of additional SBS improvement at 5 wt.% nFAp becomes particularly interesting when considered together with the microstructural findings. The 5 wt.% formulation showed the highest measured porosity and resolved pore number density, while BSE-SEM/EDS demonstrated local compositional heterogeneity. One possible explanation is therefore that the beneficial effects associated with nFAp incorporation become partly counterbalanced at higher filler content by a greater contribution of structural discontinuities. Pores located within highly stressed regions of the cement layer may facilitate crack initiation and thus limit the improvement in macroscopic bond strength. Such a mechanism would be consistent with the observation that 5 wt.% nFAp retained significantly higher SBS than REF but did not outperform the 2 wt.% formulation. This remains a hypothesis, particularly because the micro-CT comparison was based on one specimen per formulation and should be verified using replicated structural analyses and direct examination of fracture paths.

Weibull analysis provided complementary information on bond-strength reliability. The 2 wt.% formulation showed the most favorable descriptive Weibull profile, suggesting a more consistent bond-strength distribution. However, because conventional statistical analysis did not demonstrate a significant difference between the two modified formulations, these findings should not be interpreted as evidence of statistical superiority of 2 wt.% nFAp. The ARI findings provide an important complement to the SBS results. Despite the significant increase in bond strength after nFAp incorporation, the distribution of ARI scores did not differ significantly among the groups [44]. Thus, the higher SBS of the modified cements was not accompanied by a detectable shift in the macroscopic failure pattern. However, ARI provides only an ordinal estimate of the amount of cement remaining on enamel and does not describe the actual crack path through the bonded system. Montasser and Drummond demonstrated that ARI classification may change with the magnification used for assessment and also discussed evidence that qualitative ARI scores do not closely reproduce quantitative measurements of the residual adhesive area [45,46].

From a mechanical perspective, this distinction is particularly relevant because a nominal SBS test does not produce a uniform shear-stress field. Algera et al. [47] demonstrated using finite element analysis that stresses within the bracket–cement–enamel system are highly non-uniform and that fracture initiates at local peak-stress regions. Their fractographic observations also showed that failure under shear loading may evolve through both adhesive and cohesive regions rather than following a single interface throughout the entire bonded area [47]. They further noted that initial defects such as voids or cracks may act as preferential sites of fracture initiation. In their shear-loaded specimens, crack development involved a complex transition between interfacial and cohesive failure as the loading conditions changed during debonding.

Taken together, the SBS and ARI results suggest a more complex failure process. Two specimens assigned to the same ARI category may have reached the same final macroscopic distribution of residual cement while undergoing different crack-initiation and propagation processes. Consequently, the significant increase in SBS without a corresponding shift in ARI distribution may indicate that nFAp affected the resistance of the bonded system to crack initiation or propagation rather than changing the final macroscopic location of failure. Such an effect could occur without a measurable increase in local indentation stiffness or hardness. This interpretation remains hypothetical, but it provides a mechanically plausible explanation for the present findings and identifies a relevant direction for further work. Direct fractographic examination of both sides of the debonded interface, high-resolution mapping of crack paths, and fracture-mechanics-based testing would be required to determine whether nFAp modifies the balance between interfacial and cohesive crack propagation.

Interpretation of the ARI results also requires consideration of the experimental geometry. In the present study, scoring was based exclusively on the amount of cement remaining on enamel. The material visible on the debonded tube base was considered descriptive because its quantity depends not only on fracture location but also on the amount of cement initially placed around the base, marginal extrusion during tube placement, adaptation of the tube to the curved buccal enamel surface, and local cement-layer thickness. Consequently, the amount of material remaining on the tube cannot be regarded as a direct complementary measure of ARI. Quantitative image segmentation of residual cement on enamel would provide a more objective assessment in future studies and could be combined with profilometry or confocal microscopy to evaluate surface changes after cleanup [45,48,49].

Several limitations should be considered. The study was performed in vitro and therefore did not reproduce the combined effects of saliva, fluctuating pH, biofilm exposure, thermocycling, cyclic masticatory loading, and long-term hydrothermal ageing. Consequently, the present SBS results should not be interpreted as evidence of long-term clinical performance in the oral environment. In addition, the study did not include a conventional resin-based orthodontic adhesive as a separate control; therefore, the present results do not allow direct comparison of the nFAp-modified RMGIC with resin-based orthodontic bonding systems used in clinical practice [50]. Micro-CT was performed on one physical specimen per formulation; the three Ø2 mm regions therefore represent spatial sampling within a specimen rather than independent material replicates. Reconstruction-related ring artifacts required the local quantitative analysis to be restricted to artifact-free regions, and the voxel size of 5.93 µm did not permit direct visualization of individual nFAp nanoparticles. EDS was semiquantitative and performed on fractured surfaces, while the indentation analysis included three specimens per formulation and intentionally sampled visually defect-free regions. SBS analysis was based on 28 valid specimens, and ARI remained a qualitative ordinal assessment. These limitations should be considered when extrapolating the present mechanistic interpretation. The limited number of independent specimens, particularly in the indentation analysis (*n* = 3 per formulation), also reduced the statistical power necessary to detect small or moderate between-group differences. Consequently, nonsignificant findings for instrumented indentation analysis and ARI should not be interpreted as evidence of equivalence or absence of an effect. These outcomes should instead be regarded as preliminary estimates that require confirmation in studies with larger numbers of independent specimens.

Taken together, the present findings suggest that the effect of nFAp on orthodontic bonding cannot be explained by a simple increase in bulk hardness or stiffness. The observed response is more consistent with a complex interplay between cement microstructure, local composition, and the mechanics of the bonded interface. Direct interfacial and fractographic studies will be required to determine which of these factors contributes most strongly to the increased resistance to debonding.

## 5. Conclusions

Incorporation of nanofluorapatite into GC Fuji Ortho LC significantly improved shear bond strength, with increases of approximately 39% and 34% for the 2 wt.% and 5 wt.% formulations, respectively, compared with the unmodified cement. The 2 wt.% formulation showed the highest mean SBS and the most favorable descriptive Weibull profile, while increasing the nFAp content to 5 wt.% did not provide any further significant improvement in bond strength.

The enhanced SBS was not accompanied by significant changes in indentation hardness, indentation modulus, or ARI distribution. At the same time, microstructural analysis showed increased porosity, higher resolved pore number density, and local compositional heterogeneity in the nFAp-modified materials, together with a markedly higher local Ca signal. These findings indicate that the improved bonding performance cannot be explained by simple bulk reinforcement of the cement matrix and likely reflects a more complex interaction between material structure and the bonded tooth–cement–orthodontic tube system.

Overall, the relationship between nFAp content and bonding performance appears to be non-linear, with 2 wt.% nFAp showing the most favorable descriptive overall mechanical profile among the tested formulations. Further studies should include replicated microstructural analysis, ageing and fatigue loading, direct characterization of the cement–enamel interface, and fractographic evaluation of crack initiation and propagation to clarify the mechanisms responsible for the observed improvement in bond strength.

## Figures and Tables

**Figure 1:**
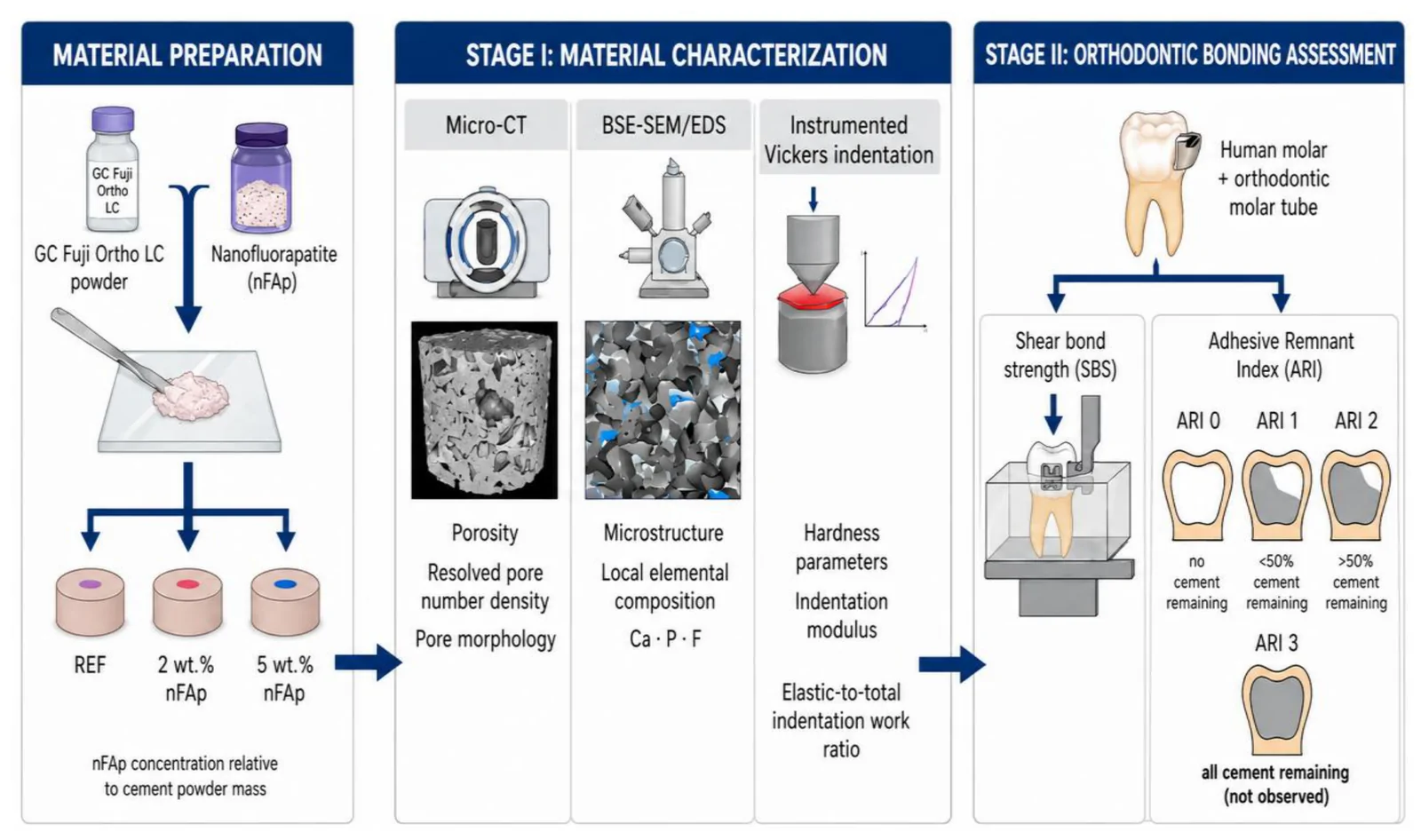
Graphical overview of the experimental workflow, including preparation of unmodified and nanofluorapatite-modified GC Fuji Ortho LC formulations, material characterization by micro-computed tomography, BSE-SEM/EDS, and instrumented Vickers indentation, followed by assessment of orthodontic molar tube bonding using shear bond strength testing and Adhesive Remnant Index scoring.

**Figure 2:**
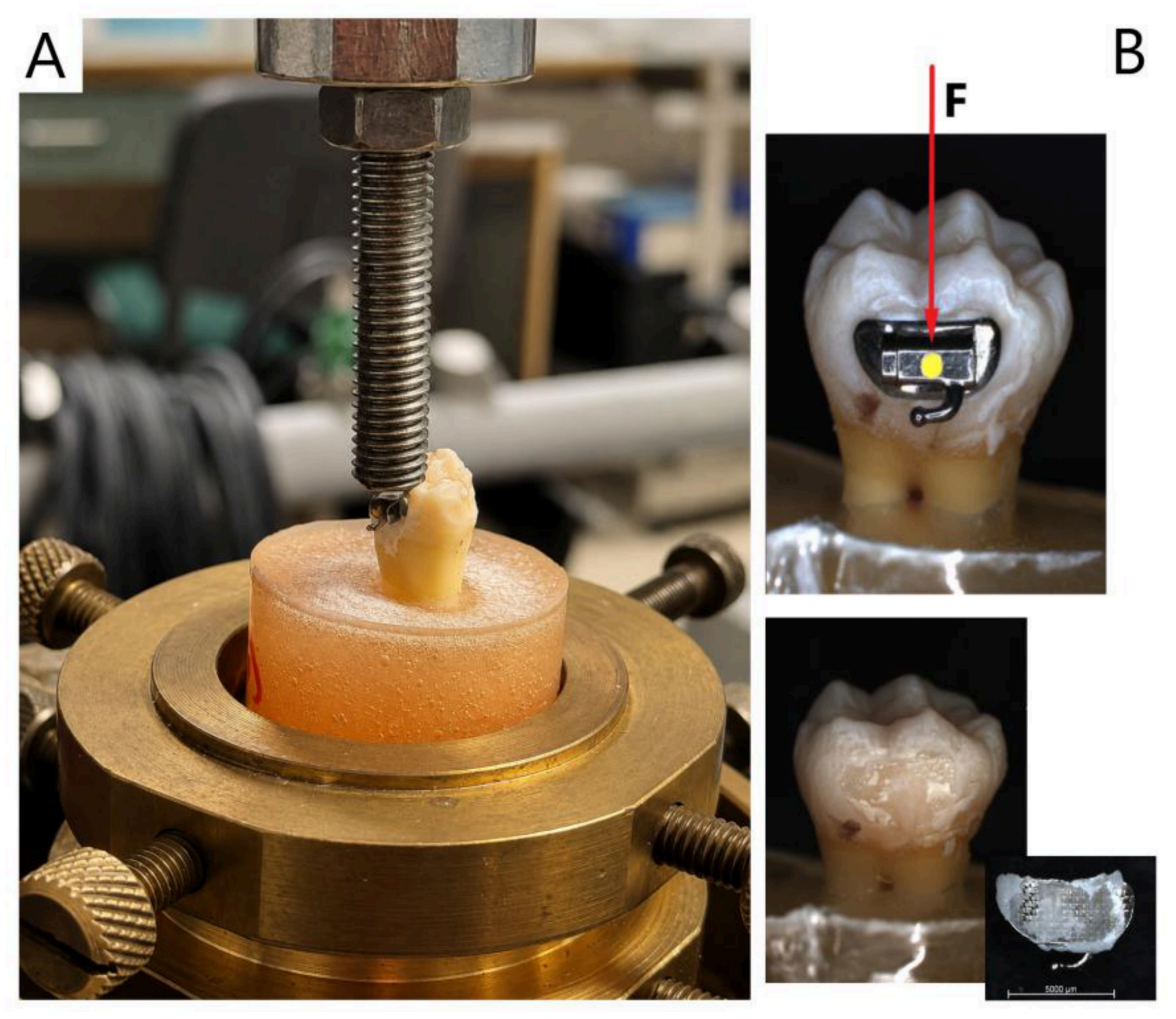
Experimental setup for shear bond strength testing of orthodontic molar tubes bonded to human enamel. (**A**) Tooth specimen embedded in self-polymerizing acrylic resin and mounted in the MTS 858 MiniBionix testing system, with the loading punch positioned adjacent to the orthodontic tube. (**B**) Direction of the applied shear force (F) along the tube–enamel interface, together with representative views of the tooth and orthodontic tube after debonding.

**Figure 3:**
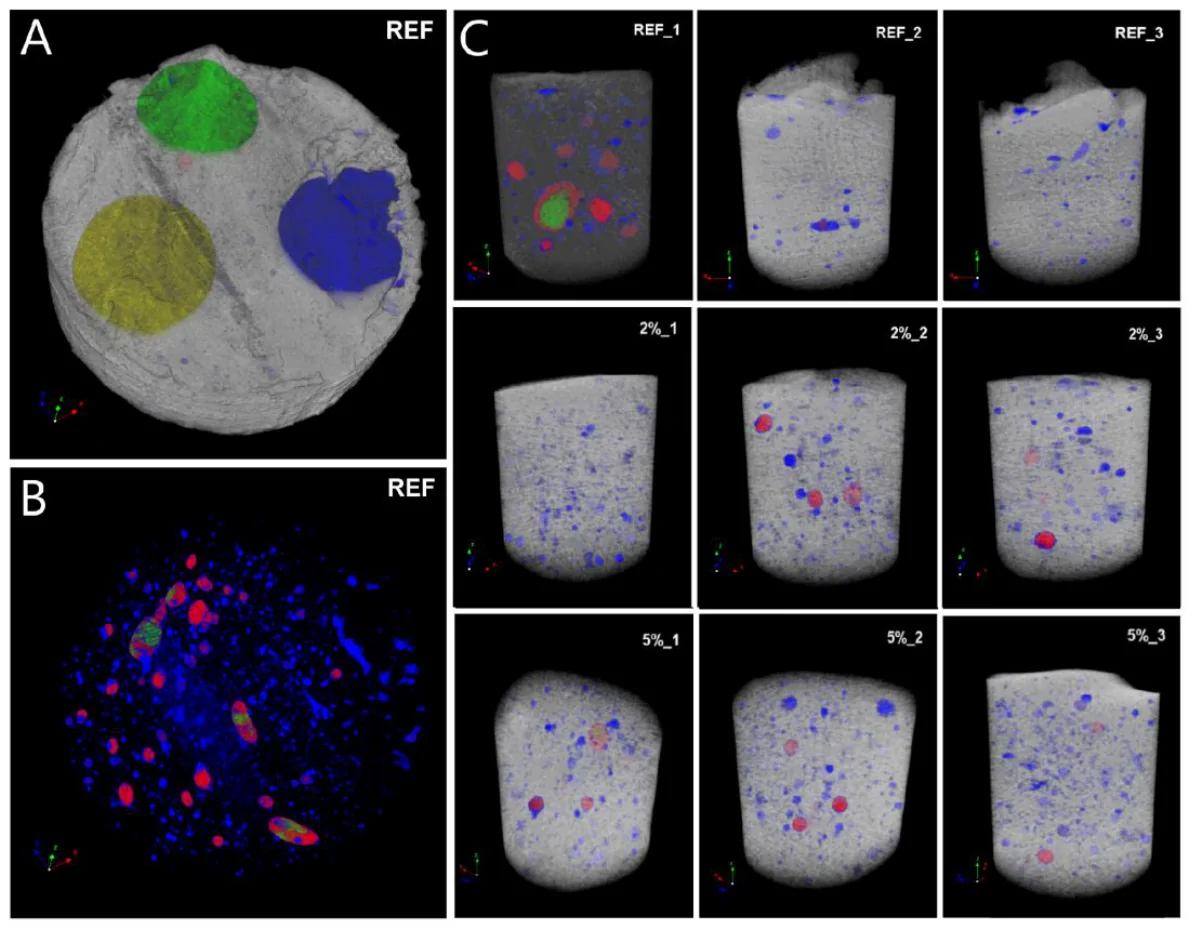
Representative micro-CT visualizations of the internal pore structure of GC Fuji Ortho LC and nFAp-modified formulations. (**A**) Three-dimensional reconstruction of the reference specimen showing three spatially separated Ø2 mm regions of interest (ROIs) used for quantitative analysis; green, yellow, and purple indicate the selected ROIs. (**B**) Segmented internal pore network of the reference specimen. Pores are color-coded by size: blue—small, red—medium, and green—large. (**C**) Representative three-dimensional reconstructions of the three Ø2 mm ROIs analyzed for REF, 2 wt.% nFAp, and 5 wt.% nFAp formulations. Colored overlays indicate segmented pores according to the same size classification. Scale bars: 2 mm in (**A**) and 500 µm in (**C**).

**Figure 4:**
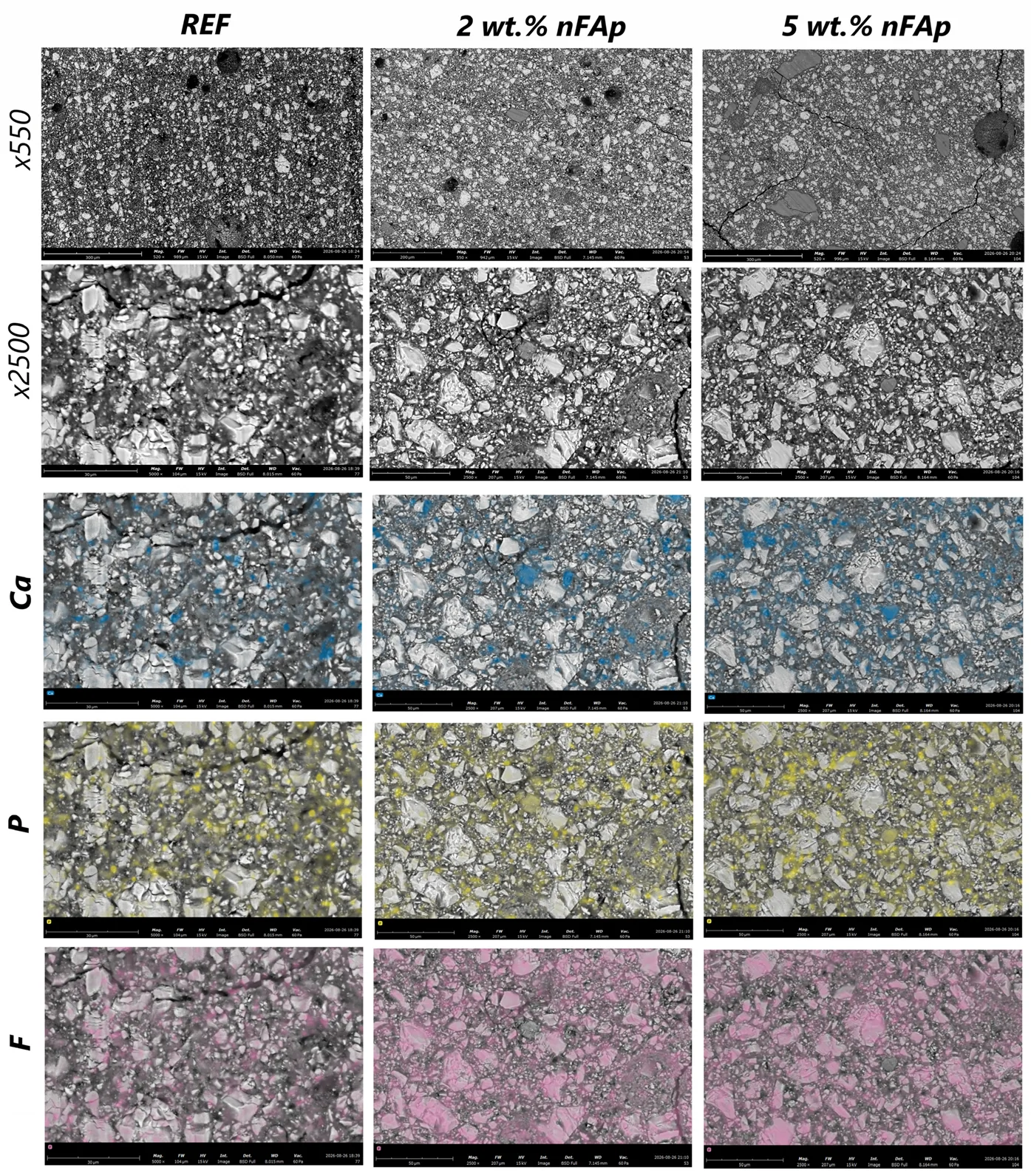
Representative backscattered-electron scanning electron microscopy (BSE-SEM) images and corresponding energy-dispersive X-ray spectroscopy (EDS) elemental maps of the investigated cement formulations. Columns represent unmodified GC Fuji Ortho LC (REF), GC Fuji Ortho LC containing 2 wt.% nFAp, and GC Fuji Ortho LC containing 5 wt.% nFAp. BSE-SEM images are shown at ×550 and ×2500 magnification. The corresponding EDS maps obtained from the ×2500 fields show the spatial distribution of Ca, P, and F within the cement microstructure. Colored overlays indicate the detected elemental distribution.

**Figure 5:**
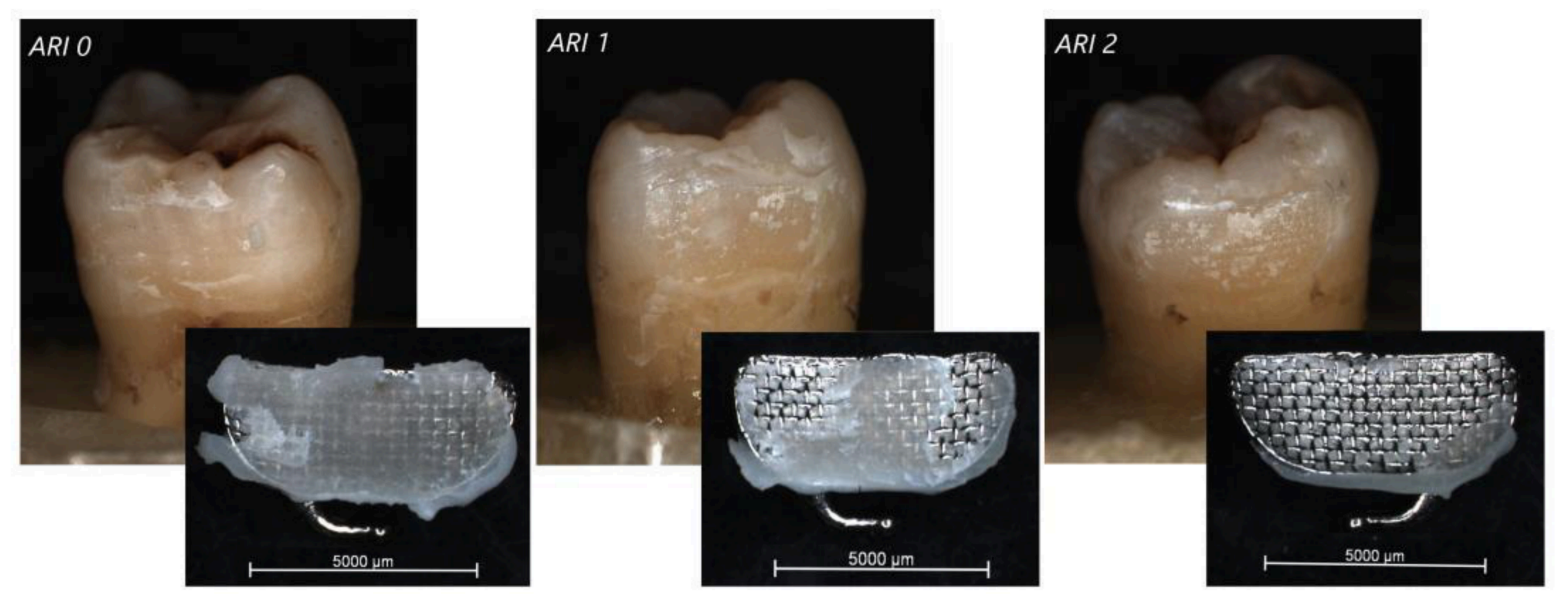
Representative stereomicroscopic images illustrating the Adhesive Remnant Index (ARI) categories observed after debonding of G&H^®^ VIPER™ orthodontic molar tubes from human enamel. The upper images show the buccal enamel surfaces after debonding, whereas the corresponding lower insets show the debonded molar tube bases. ARI 0: no cement remained on the enamel surface; ARI 1: less than 50% of the cement remained on the enamel surface; ARI 2: more than 50% of the cement remained on the enamel surface. ARI 3 was not observed in the analyzed specimens. Images of the molar tube bases are shown for descriptive purposes only and were not used for ARI classification. Scale bars: 5000 µm.

**Table 1:** Micro-CT-derived porosity parameters of the investigated cement formulations.

Parameter	REF	2 wt.% nFAp	5 wt.% nFAp
Porosity, Ø2 mm regions [%]	3.14 ± 1.12	5.05 ± 0.80	5.53 ± 0.80
Whole-specimen porosity [%]	4.09	5.06	5.40
Resolved pore number density, Ø2 mm regions [mm^−3^]	968 ± 153	1189 ± 100	1252 ± 23
Whole-specimen resolved pore number density [mm^−3^]	887	1012	1076
Resolved pore-volume fraction, Ø2 mm regions [%]	80.35 ± 6.46	88.24 ± 0.89	89.01 ± 1.96
Whole-specimen resolved pore-volume fraction [%]	87.57	90.08	90.67

Values expressed as mean ± SD were calculated from three spatially separated Ø2 mm regions and represent within-specimen spatial variability. Whole-specimen values are presented as complementary descriptive measurements.

**Table 2:** Morphometric characteristics of resolved pores in the pooled Ø2 mm regions and whole specimens.

Parameter		REF	2 wt.% nFAp	5 wt.% nFAp
ESD, median (Q1–Q3) (µm)	Ø2 mm pooled	25.99 (22.35–31.85)	27.30 (23.12–34.51)	27.12 (22.87–34.51)
	Whole-specimen	26.75 (22.87–33.11)	27.30 (22.87–34.62)	27.48 (23.12–35.06)
ESD, mean (µm)	Ø2 mm pooled	28.94 ± 12.21	31.29 ± 15.27	31.35 ± 15.63
	Whole-specimen	30.06 ± 13.92	31.47 ± 16.07	31.79 ± 16.11
Feret Min, median (Q1–Q3) (µm)	Ø2 mm pooled	23.73 (20.32–29.67)	23.73 (20.97–33.56)	23.90 (21.00–35.11)
	Whole-specimen	23.73 (20.66–30.52)	23.73 (20.98–33.61)	24.41 (21.04–35.39)
Feret Mean, median (Q1–Q3) (µm)	Ø2 mm pooled	37.64 (30.96–48.83)	39.66 (32.06–53.39)	39.54 (31.77–54.00)
	Whole-specimen	38.49 (31.32–50.81)	39.57 (31.86–53.26)	39.82 (31.94–54.46)
Feret Max, median (Q1–Q3) (µm)	Ø2 mm pooled	53.15 (41.95–71.67)	56.29 (43.59–79.59)	54.75 (42.78–76.17)
	Whole-specimen	54.75 (42.78–75.47)	56.24 (43.19–78.21)	54.75 (42.78–77.02)

Morphometric parameters were calculated for resolved pores identified within the pooled three Ø2 mm regions. Whole-specimen values are shown for comparison. Resolved pores were defined as connected components with a minimum Feret diameter ≥ 17.80 µm.

**Table 3:** Semiquantitative EDS composition of selected elements in the investigated cement formulations.

Element [at.%]	REF (*n* = 4)	2 wt.% nFAp (*n* = 3)	5 wt.% nFAp (*n* = 3)
F	14.92 ± 0.90	13.42 ± 2.97	15.18 ± 1.00
P	0.554 ± 0.032	0.574 ± 0.060	0.553 ± 0.080
Ca	0.096 ± 0.020	0.267 ± 0.202	0.257 ± 0.067

Values are presented as mean ± SD of specimen-level means. Three EDS spot measurements were averaged within each specimen. Specimen 87 from the 5 wt.% nFAp group was excluded from quantitative EDS comparison because of Au coating.

**Table 4:** Instrumented indentation parameters of the investigated cement formulations.

Parameter	REF	2 wt.% nFAp	5 wt.% nFAp	*p*-Value
HM [MPa]	572.64 ± 25.65	615.17 ± 129.78	621.32 ± 81.92	0.651
H_IT_ [MPa]	751.84 ± 27.35	832.82 ± 216.39	835.07 ± 135.30	0.609
E_IT_ [GPa]	20.17 ± 2.00	19.82 ± 1.30	20.10 ± 1.42	0.962
HV_IT_ [HV]	70.96 ± 2.58	78.60 ± 20.42	78.82 ± 12.77	0.609
η_IT_ [%]	25.50 ± 0.78	25.62 ± 4.55	26.10 ± 1.29	0.827

Values are presented as mean ± SD based on three independent specimens per formulation. Individual indentation measurements were averaged within each specimen before statistical analysis. *p*-values were determined using Welch’s one-way ANOVA.

**Table 5:** Shear bond strength of orthodontic molar tubes bonded using the investigated cement formulations.

Group	*n*	SBS [MPa]	Median [MPa]	95% CI [MPa]	Weibull β	Weibull η [MPa]
REF	10	12.47 ± 2.41 ^a^	11.80	10.75–14.20	5.993	13.448
2 wt.% nFAp	9	17.31 ± 2.34 ^b^	16.37	15.51–19.11	8.199	18.306
5 wt.% nFAp	9	16.66 ± 2.60 ^b^	16.08	14.66–18.66	7.115	17.746

Values are presented as mean ± SD. Different superscript letters indicate statistically significant differences between groups according to Tukey’s HSD test (*p* < 0.05). CI, confidence interval; β, Weibull shape parameter; η, Weibull scale parameter.

**Table 6:** Distribution of Adhesive Remnant Index (ARI) scores among the investigated cement formulations.

Group	*n*	ARI 0	ARI 1	ARI 2	ARI 3
REF	10	3 (30.0%)	5 (50.0%)	2 (20.0%)	0
2 wt.% nFAp	9	3 (33.3%)	5 (55.6%)	1 (11.1%)	0
5 wt.% nFAp	9	1 (11.1%)	5 (55.6%)	3 (33.3%)	0

Values are presented as number of specimens (%). Differences in ARI score distribution among the groups were evaluated using the Fisher–Freeman–Halton exact test.

## Data Availability

The original contributions presented in this study are included in the article. Further inquiries can be directed to the corresponding authors.
